# HIV-1 encoded candidate micro-RNAs and their cellular targets

**DOI:** 10.1186/1742-4690-1-43

**Published:** 2004-12-15

**Authors:** Yamina Bennasser, Shu-Yun Le, Man Lung Yeung, Kuan-Teh Jeang

**Affiliations:** 1 Molecular Virology Section, Laboratory of Molecular Microbiology, National Institute of Allergy and Infectious Diseases, National Institutes of Health, Bethesda, Maryland 20892-0460, USA; 2 Laboratory of Experimental and Computational Biology, National Cancer Institute Center for Cancer Research, National Cancer Institute, Frederick, MD 21702, USA

## Abstract

MicroRNAs (miRNAs) are small RNAs of 21–25 nucleotides that specifically regulate cellular gene expression at the post-transcriptional level. miRNAs are derived from the maturation by cellular RNases III of imperfect stem loop structures of ~ 70 nucleotides. Evidence for hundreds of miRNAs and their corresponding targets has been reported in the literature for plants, insects, invertebrate animals, and mammals. While not all of these miRNA/target pairs have been functionally verified, some clearly serve roles in regulating normal development and physiology. Recently, it has been queried whether the genome of human viruses like their cellular counterpart also encode miRNA. To date, there has been only one report pertaining to this question. The Epstein-Barr virus (EBV) has been shown to encode five miRNAs. Here, we extend the analysis of miRNA-encoding potential to the human immunodeficiency virus (HIV). Using computer-directed analyses, we found that HIV putatively encodes five candidate pre-miRNAs. We then matched deduced mature miRNA sequences from these 5 pre-miRNAs against a database of 3' untranslated sequences (UTR) from the human genome. These searches revealed a large number of cellular transcripts that could potentially be targeted by these viral miRNA (vmiRNA) sequences. We propose that HIV has evolved to use vmiRNAs as a means to regulate cellular milieu for its benefit.

## Findings

Initially discovered in *Caenorhabditis elegans *as regulators of temporal control of post-embryonic development [1,2], miRNAs are small RNAs involved in the specific regulation at the post-transcriptional level of cellular genes in various organisms such as flies, plants and mammals [3,4]. To date, more than two hundred human miRNAs have been described [5]. Structurally, miRNAs are 21 to 25 nucleotide RNAs derived from the maturation of a hairpin precursor transcript which can be encoded by the 3' untranslated region of genes, introns of genes, or by specific chromosomal regions composed of tandem clusters of miRNA sequences. Precursor RNAs for miRNAs are structured as imperfect RNA hairpins containing mismatches and bulges. In mammalian cells, the maturation of miRNA occurs in two steps consecutively involving two cellular RNase III proteins, the nuclear Drosha and the cytoplasmic Dicer [6]. Accordingly, a miRNA precursor is specifically recognized in the nucleus by Drosha which cleaves the RNA to release an imperfect stem-loop structure of ~ 70 nucleotides, the pre-miRNA. This structure is then exported by exportin-5 into the cytoplasm and further cleaved there by Dicer into corresponding imperfect RNA duplexes of 21 to 25 nucleotides, the miRNA [7]. Mechanistically, either one of the two strands of the mature miRNA can be incorporated into the RNA-induced silencing complex (RISC). miRNA-armed RISCs can then specifically recognize and interact via imperfect complementarity with RNA targets to induce repression of translation and (less frequently) mRNA cleavage. The precise molecular mechanism of translational silencing remains unclear; however, in such instances, it has been observed that protein synthesis is inhibited while the stability of the mRNA is not altered [8-10].

Recently, in addition to plants, insects, invertebrate animals, and mammals, Pfeffe*r et al*. identified virus-encoded miRNA sequences in Epstein-Barr virus (EBV) infected cells [11]. They reported that EBV encodes five miRNAs each capable of regulating viral genes involved in latency as well as modulating the expression of host cell genes. Thus, it would appear that EBV has evolved to use the miRNA pathway for its replicative benefit. To query whether this stratagem might also be employed by other viruses, we have analyzed putative miRNA-encoding capacity of HIV-1.

**Figure 1:**
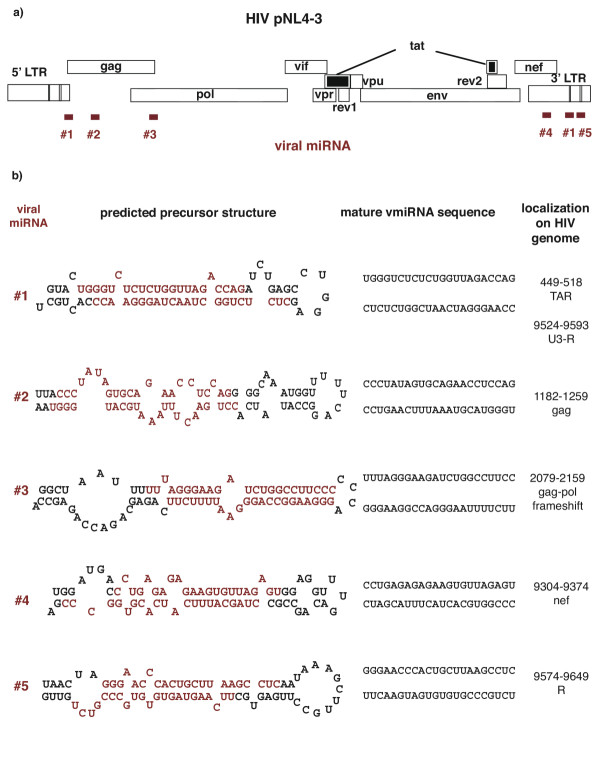
**Sequences and localization of HIV-encoded miRNA candidates. a) **Locations for 5 predicted pre-miRNAs candidates in the pNL4-3 genome are shown. **b**) The folded structures of the 5 viral pre-miRNAs from pNL4-3 (Accession Number AF 324493) [17] are illustrated. Folded pre-miRNAs and their corresponding predicted mature viral miRNA (red) are listed. Nucleotide positions (where 1 is the initiation of transcription) in the pNL4-3 genome are presented in the right column.

We wondered if HIV-1 maintains RNA structures that resemble pre-miRNAs. As a proof-of-principle, we examined pre-miRNA structures in one specific example of HIV-1, the genome of the pNL4-3 molecular clone. Because HIV has well-described stem-loops such as TAR (trans-activation responsive RNA) and RRE (Rev-responsive element) [12], one might think that pre-miRNA structures would be prevalently found in this virus. However, when we set search parameters to include RNA structure of ~ 70 nucleotides in total size with an imperfect stem of 21 to 25 base-pairs, only a few thermodynamically reasonable candidates were revealed. Using a new scanning method *StemEd *[13], we uncovered 5 pre-miRNA candidates. As shown in figure 1a, these sequences (#1 to #5) are discretely separated in different regions of the HIV genome: near TAR, in capsid *gag*, near the *gag-pol *frameshift, in the *nef *gene, and in the 3'LTR [14,15]. The corresponding predicted folding for each candidate and their deduced mature virally-encode miRNA (vmiRNA) sequences are presented in figure 1b.

**Figure 2:**
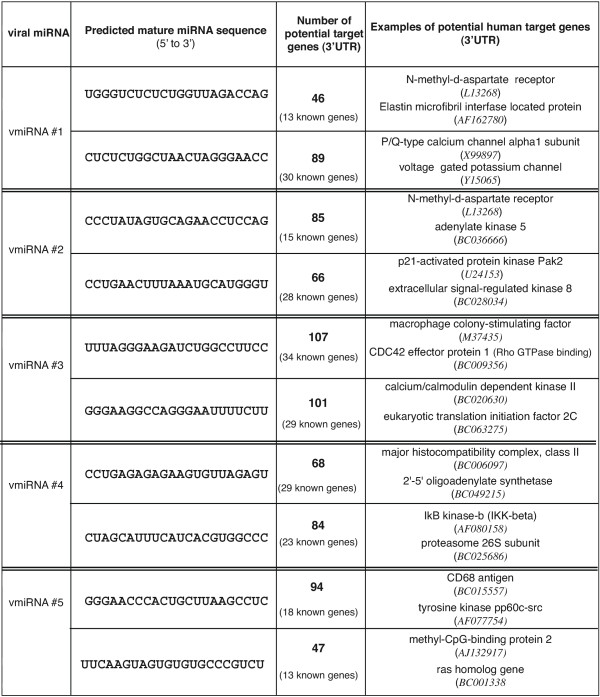
**Potential cellular targets for each of the vmiRNAs. **The two deduced mature vmiRNAs predicted from each precursor miRNA are shown. The mature vmiRNA sequences were individually searched against a database of human 3'UTRs using imperfect complementarity criteria as described in the text. The number of potential candidate cellular RNA targets is enumerated. Most of the cellular targets are incompletely characterized expressed sequence tag (EST) clones, with a subset of targets being known genes. For each predicted vmiRNA, we list two examples of known cellular gene targets at the right. A full list of targets is available upon request.

The 5 HIV-1 encoded pre-miRNA candidates can in principle yield 10 mature vmiRNAs. To ask, whether these putative vmiRNAs, if expressed in infected cells, could be used by HIV-1 to modulate host cell gene expression profiles (i.e. suppress the translatability of cellular mRNAs), we checked each vmiRNA sequence against a 3'UTR database for human genes. Because the exact rules governing suppression of translation based on miRNA complementarity to 3' UTR remain unclear, we collected all "hits" that had 6 or fewer mismatches with an additional constraint that the 5'-most nucleotide of the vmiRNA cannot be mis-matched with the target sequence. Surprisingly, based on the above criteria, a very large number of cellular targets were found (Figure 2). On average, it is suggested that each vmiRNA could target 50 to 100 cellular RNAs. If all 10 vmiRNAs were functionally competent, this would argue that HIV-1 could potentially modulate the expression of 500 to 1,000 cellular transcripts using this mechanism. Intriguingly, in the setting where a limited number of mis-matches are permitted, the same vmiRNA apparently can target functionally distant cellular proteins such as IkB-kinase-β and proteasome 26S subunit or macrophage colony-stimulating factor M-CSF and CDC42 effector protein 1 (Figure 2). This suggests that vmiRNAs could pleiotropically affect the expression pattern of cellular proteins.

Here, we introduce the concept that the HIV genome could reasonably encode 5 candidate pre-miRNAs. We further suggest that a large number of cellular transcripts could potentially be targeted if these 5 pre-miRNAs were processed into 10 predicted mature vmiRNAs (Figure 2). Studies are in progress to verify experimentally the expression of our candidate vmiRNAs in HIV-1 infected cells. If HIV-1 encoded vmiRNA candidates can be shown to be functional, their action could, in part, explain the frequently observed landscape changes in host cell gene expression profiles during HIV-1 infection as revealed by micro-array studies [16]. We are also currently examining how vmiRNAs might additionally affect HIV-1 gene expression.

## Competing interests

The authors declare that they have no competing interests.

## Authors' contributions

YB and KTJ conceived of the ideas and wrote the paper. SYL did the computation work for the study. MLY participated in the discussion of the data.
